# Invasive pulmonary aspergillosis in critically ill patients with severe COVID-19 pneumonia: Results from the prospective AspCOVID-19 study

**DOI:** 10.1371/journal.pone.0238825

**Published:** 2021-03-17

**Authors:** Tobias Lahmer, Silja Kriescher, Alexander Herner, Kathrin Rothe, Christoph D. Spinner, Jochen Schneider, Ulrich Mayer, Michael Neuenhahn, Dieter Hoffmann, Fabian Geisler, Markus Heim, Gerhard Schneider, Roland M. Schmid, Wolfgang Huber, Sebastian Rasch

**Affiliations:** 1 Klinik und Poliklinik für Innere Medizin II, Klinikum rechts der Isar der Technischen Universität München, Munich, Germany; 2 Klinik und Poliklinik für Aneasthesiologie und Intensivmedizin, Klinikum rechts der Isar der Technischen Universität München, Munich, Germany; 3 Institut für Mikrobiologie, Immunologie und Hygiene, Technische Universität München, Munich, Germany; 4 Institut für Virologie, Technische Universität München, Munich, Germany; National and Kapodistrian University of Athens, GREECE

## Abstract

**Background:**

Superinfections, including invasive pulmonary aspergillosis (IPA), are well-known complications of critically ill patients with severe viral pneumonia. Aim of this study was to evaluate the incidence, risk factors and outcome of IPA in critically ill patients with severe COVID-19 pneumonia.

**Methods:**

We prospectively screened 32 critically ill patients with severe COVID-19 pneumonia for a time period of 28 days using a standardized study protocol for oberservation of developement of COVID-19 associated invasive pulmonary aspergillosis (CAPA). We collected laboratory, microbiological, virological and clinical parameters at defined timepoints in combination with galactomannan-antigen-detection from nondirected bronchial lavage (NBL). We used logistic regression analyses to assess if COVID-19 was independently associated with IPA and compared it with matched controls.

**Findings:**

CAPA was diagnosed at a median of 4 days after ICU admission in 11/32 (34%) of critically ill patients with severe COVID-19 pneumonia as compared to 8% in the control cohort. In the COVID-19 cohort, mean age, APACHE II score and ICU mortality were higher in patients with CAPA than in patients without CAPA (36% versus 9.5%; p<0.001). ICU stay (21 versus 17 days; p = 0.340) and days of mechanical ventilation (20 versus 15 days; p = 0.570) were not different between both groups. In regression analysis COVID-19 and APACHE II score were independently associated with IPA.

**Interpretation:**

CAPA is highly prevalent and associated with a high mortality rate. COVID-19 is independently associated with invasive pulmonary aspergillosis. A standardized screening and diagnostic approach as presented in our study can help to identify affected patients at an early stage.

## Introduction

Since the outbreak of the novel severe acute respiratory syndrome coronavirus 2 (SARS-CoV-2)–associated respiratory disease in December 2019, numerous patients were hospitalized with viral pneumonia and respiratory insufficiency, which was finally designated as the clinical coronavirus disease 2019 (COVID-19) [1]. Nearly 5% of the affected COVID-19 patients are critically ill, develop an acute respiratory distress syndrome (ARDS) and need intensive care unit management including mechanical ventilation [1, 2].

Along with other uncertainties during an intensive care unit (ICU) stay, superinfections, including invasive pulmonary aspergillosis (IPA), are well-known complications of severe viral pneumonia in critically ill patients. First reported in the 1950´s the association between viral pneumonia and IPA was reported in a greater cohort during the H1N1 influenza seasons 2009–2011 by Wauters et al. [3, 4]. Surprisingly, not only the rate of IPA was higher than suspetced (incidence of 23%) but also nearly half of the IPA patients did not fulfill the classical risk factors of the European organisation for research and tretament of cancer/ mycosis study group (EORTC/MSG) for IPA developement in this cohort [5]. These findings could be confirmed by the dutch-belgian mycosis study group and resluted not only in the recognition of influenza as an independent risk factor for IPA devlopement but also in modified definitions and diagnostic criterias for IPA in critically ill patients [6, 7].

With the (modified) AspICU algorithm for critically ill patients were adapted diagnostic criterias for IPA could be established beside the EORTC/MSG criteria [8]. However, the new clinical conditions of COVID-19 patients along with infection control restrictions for biosampling will make the diagnostic procedures and microbiological interpretation for IPA in COVID-19 patients even more challenging.

In analogy to the experiences in critically ill patients with severe influenza associated pneumonia, the aim of our prospective AspCOVID-19 study is to describe the incidence and outcome of COVID-19 associated invasive pulmonary aspergillosis (CAPA) in critically ill patients with severe pneumonia using a standardized screening procedure and assess whether COVID-19 is independently associated with IPA.

## Methods

### Study design and participants

This prospective cohort study was conducted at two tertiary care ICU´s (department of internal medicine and department of anaesthesiology) of the University Hospital of the Technical University of Munich, Germany.

Between March and April 2020, 32 patients with severe COVID-19 associataed pneumonia were prospectively included in the AspCOVID-19 study.

Adult patients (18 years of age or older) with confirmed severe COVID-19 pneumonia (clinical signs, typical laboratory constellation, PCR test for SARS-CoV-2 positive and chest computed tomography (CT) scan with typical signs) who were admitted to the ICU due to acute respiratory failure for more than 48 hours with the need for mechanical ventilation were eligible for study inclusion.

64 COVID-19 negative critically ill adult patients with ARDS and pneumonia without immunosuppression were included as a retrospective matched control group. Selection criteria for the control cohort were ARDS caused by pneumonia with a corresponding Horowitz Index <150 mmHg as well as comparable sequential organ failure assessment (SOFA) and acute physiology and chronic health evaluation (APACHE II) scores. Exclusion criteria of the COVID-19 as well the control cohort were as follows:

- Patients fulfilling the EORTC/MSG criteria
- Immunosuppression
- mycological evidence from only one specimen from lower repiratory tract and no correlation in broncho-alveolar lavage (BAL) or standard microbiological findings (control cohort)
- Not fulfilling of the adapted AspICU /CAPA definitions (COVID-19 cohort)

A total of 347 ICU patients, from 01/2018 till 12/2020, were screened for the retrospective machted control cohort, 283 were excluded (215 did not fulfill the selection criteria, 67 met the EORTC/MSG criteria, 1 patient was *Aspergillus spp*. colonized). Pregnancy, age younger than 18 years, insufficient available information or lacking written informed consent were general exclusion criterias.

Only patients fulfilling the inclusion/exclusion criteria for the COVID-19 as well for the control cohort were included in this study.

The study was approved by the institutional review board, Klinikum rechts der Isar, TU München (Ref. 149/20S) and registred as a prospective study at ISRCTN (https://doi.org/10.1186/ISRCTN14810048). Written informed consent was obtained by the patient or their legal representatives.

### Screening for aspergillosis of critically ill patients with COVID-19 and control cohort

**Fig 1:**
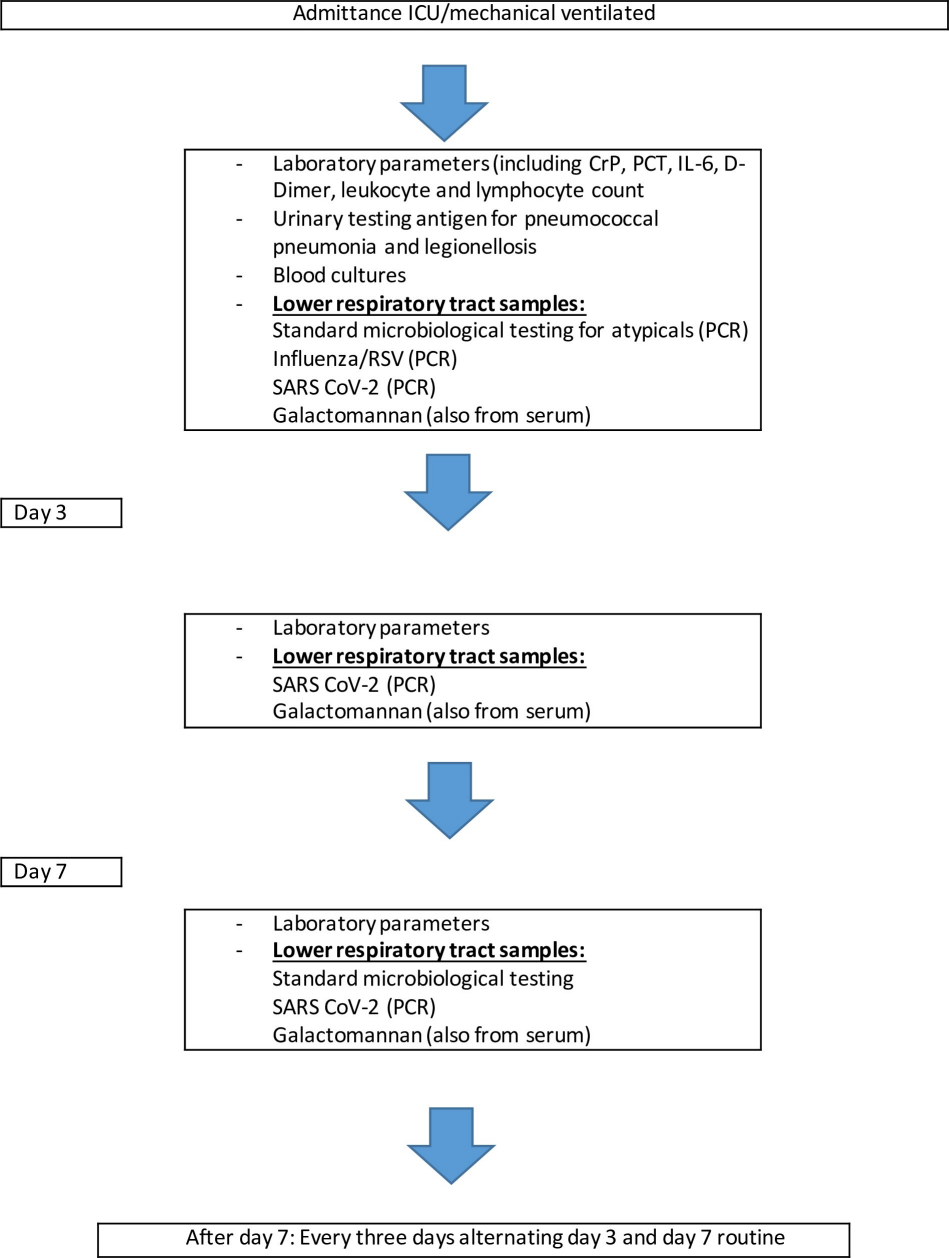
Standardized diagnostic algorithm for patients with suspected or confirmed COVID-19 associated ARDS.

Patients of the COVID-19 cohort were prospectively screened in defined time intervals for developement of Covid-19 associated invasive pulmonary aspergillosis (CAPA) following the study protocol **(**Fig 1**)**.

In addition standard microbiological, virological and laboratory tests were performed at time intervals summarized in the study protocol (at admission tests were also perforemd before ICU admittance in the emergency department or general ward. If these tests were performed >1 day before ICU admission all test were repeated at ICU admittance). All patients received a chest CTscan before ICU admittance. Testing for atypical pneumonia (using PCR from bronchial aspirates, including Legionella pneumophila, Mycoplasma pneumonia and Chlamydia pneumonia, and pneumococcal antigen from urine) as well as respiratorial syncytial virus (RSV)—and influenza were performed in all patients.

For safety reasons, diagnostic testing of respiratory specimens was performed in accordance to the guidelines of the European society of intensive medicine (ESCIM) using a nondirected bronchial lavage (NBL) gained by deep bronchial suction with a closed suction system from intubated patients.

Screening for CAPA ended after extubation for all patients. In patients with positive proof of aspergillus galactomannan-antigen (GM) from respiratory and/or serum samples a serum GM follow up examination after extubation was performed. Overall ICU observation time for all COVID -19 patients was 28 days.

Results of glactomannan testing from bronchoalveolar lavage (BAL) examinations were used in the control group. These patients were screened for invasive pulmonary aspergillosis once weekly according to our local ICU standard (GM from serum and BAL). In addition, testing for atypical pneumonia (using PCR from bronchial aspirates, see above, and pneumococcal antigen from urine) as well as respiratorial syncytial virus (RSV)—and influenza were performed in all patients as in the COVID-19 cohort.

### Definitions

The modified AspICU score, developed for the diagnostic assessment of influenza associated IPA, was adapted on COVID-19 patients and used to classify IPA. Following the CAPA definitions of White PL et al., Bartoletti M et al. and van Biesen S putative IPA was assumed in one of the following conditions: cultural growth of *Aspergillus spp* from NBL speciemens.; GM optical density index (ODI) >0.5 in serum, GM ODI >1 in respiratory tract specimen from NBL [9–11]. Clinical signs and radiological signs were in line with the modified AspICU score and CAPA definitions [10, 11].

When >1 criterion necessary for CAPA diganosis was not met, these cases were classified as *Aspergillus* colonization.

Every COVID-19 patient fulfilling the mentioned criteria was discussed by a specialist (consultant) for microbiology and a specialist for intensive care medicine (consultant) to ascertain that the criterias for CAPA were appropriate.

### Microbiological and virological methods

Galactomannan (GM) detection (Platelia^TM^ Aspergilllus Ag, Bio-Rad Laboratories, Munich, Germany) was performed in serum samples and in NBL samples gained by deep tracheal suction with a closed suction system from the lower respiratory tract.

Original un-processed NBL fluid was used for cultural methods not the pellet post-processing for GM-ELISA. For microbiological culture, approx. 30μl NBL fluid were plated on each agar plate. Primary aerobic microbiological cultures from NBL fluid were performed on columbia agar and chocolate agar (prepared culture media, Becton Dickinson, Sparks, MD, United States of America). Primary cultures on agar plates were incubated for 48 h.

When growth of *Aspergillus spp*. could be established, it was then sub-cultured on sabouraud-dextrose-agar (Oxoid™ Thermo Fisher Scientific™, Waltham, MA, United States of America) (Aspergillus sub-cultures on sabouraud-dextrose-agar were incubated for 48–72 h before species identification) for species identification via macroscopic, microscopic and MALDI-TOF (Bruker Daltronics GmbH, Leipzig, Germany) analysis.

Phenotypic screening for azol-resistance in *Aspergillus spp*. was performed routinely using RPMI (Becton Dickinson, Sparks, MD, United States of America) agar plates supplemented with voriconazol (2mg/L) and itraconazol (4mg/L) and an antifungal-free agar as growth control. Agar plates for phenotypic screening for azol-resistance were incubated for 5 days. Azole susceptible isolates were identified by growth on the antifungal-free agar and absence of growth on plates containing azoles.

### Virological testing

SARS CoV-2 nucleocapsid gene was detected by Taqman RT PCR with the primers 2019-nCoV_N1-F-5`-GAC CCC AAA ATC AGC GAA AT-3`, 2019-nCoV_N1-R 5`-TCT GGT TAC TGC CAG TTG AAT CTG-3 and the probe 2019-nCoV_N1-P 5`-FAM-ACC CCG TAC GTT TGG TGG ACC-BHQ1-3`(https://www.cdc.gov/). Positive results were confirmed with another pair of N gene primers and- probe. IgG and IgM antibodies directed at SARS-CoV were detected with the iFlash 1800Chemiluminescence Immunoassay Analyzer (YHLO, China).

### Statistical analysis

Statistical analysis was performed using IBM SPSS Statistics 25 (SPSS Inc, Chicago, Illinois, USA). Samples were checked for normal distribution using the Shapiro-Wilk test. Descriptive data of normally distributed parameters are presented as mean ± standard deviation and as median and range for non-parametric parameters. The Mann-Whitney-U and Kruskas-Wallis tests were used to analyze non-parametric variables and the t-test and a one-way analysis of variances (ANOVA) to analyze variables with normal distribution. To compare qualitative parameters, chi-square test and in small samples (expected frequency of test variable less than 5) Fisher’s exact test was used. Probabilities are displayed as odds ratio (OR) with 95% confidence interval (CI). All statistical tests were two-sided with a level of significance (p-value) of 5%. Binary logistic regression models were used to identify risk factors for aspergillosis. Factors with a p-value below 0.05 in univariate analysis were included in the regression models. To control the false discovery rate after multiple testing we adjusted the level of significance to p = 0.015 by the Benjamini-Hochberg procedure for qualitative parameters in the COVID-19 positive patient cohort.

Based on previous studies, sample size was estimated as follows: Assuming 5% of invasive pulmonary aspergillosis in an overall patient population and 20% (estimated from influenza patients) in critically ill COVID-19 patients with severe pneumonia, we needed 32 patients in the COVID-19 cohort and 64 patients in the control cohort to gain a power of 0,786.

## Results

32 patients with severe COVID-19 associataed pneumonia were prospectively included in the AspCOVID-19 study. Influenza and respiriatory syncytial virus were negative in all included patients.

Basic patient characteristics of the COVID-19 cohort are summarized in Table 1. All patients in the COVID-19 cohort were SARS -CoV-2 PCR positive at ICU admission.

**Table 1:** Baseline characteristics of the COVID-19 cohort.

	COVID Cohort (n = 32)	With CAPA (n = 11)	Without CAPA (n = 21)	p value
**Baseline characteristics**				
Median age, years (range)	69.5 (27–84)	72 (58–84)	59 (27–84)	0.065
Male sex (%)	23 (72)	7 (63)	16 (76)	0.195
Mean APACHE II (SD)	18±4	22±3	17 ±3	**<0.001**
Mean SOFA (SD)	10±3	12±2	9 ±3	**0.003**
**Risk factors n (%)**				
COPD	3 (10)	2 (18)	1 (5)	0.266
Asthma	1 (3)	0 (0)	1 (5)	1.00
DM type 2	8 (25)	3 (27)	5 (24)	1.00
Art. hypertension	21 (65)	7 (63)	14 (66)	1.00
Coronary heart disease	2 (6)	1 (9)	1 (5)	1.00
CKD	5 (16)	1 (9)	4 (19)	0.637
Atrial fibrillation	5 (16)	1 (9)	4 (19)	0.637
**COVID-19 specific characteristics**				
SARS-CoV-2 PCR positive at admission	32	11	21	0.670
Symptoms before hospital admission, days (SD)	4± 2	3±1	4±2	0.080
Mean days at general ward, (SD)	2±2	1±2	7±2	0.510
Number of direct ICU admissions	20	7	13	0.235
Influenza/RSV PCR negative	32	11	21	-
Median days of fever, (range)	8 (3–12)	9 (3–15)	8 (4–13)	0.260
**ICU data**				
Mechanical ventilation	32	11	21	0.670
Mechanical ventilation days (range)	16 (3–28)	20 (8–28)	15 (3–28)	0.570
Need for vasopressors (%)	32 (100)	11 (34)	21 (66)	0.490
Renal replacement (%)	9/32 (28)	6/11 (55)	3/21 (14)	0.380
Broadspectrum antibiotics	32	11	21	0.670
**Outcome data**				
Median days of ICU stay (range)	18 (5–28)	21 (9–28)	17 (5–28)	0.222
ICU mortality (%)	6 (19)	4 (36)	2 (9,5)	**<0.001**
28 days status:				
- ICU	11	4	7	0.145
- Hospital	5	2	4	0.266
- Discharge	10	1	8	**0.025**

Abbreviations: COVID-19 associated invasive pulmonary aspergillosis (CAPA), acute physiology and chronic health evaluation (APACHE II); sequential organ failure assessment (SOFA); chronic obstructive pulmonary disease (COPD); diabetes mellitus type 2 (DM type II); chronic kidney disease (CKD); intensive care unit (ICU) standard deviation (SD); polymerase chain reaction (PCR); Intensive Care Unit (ICU); Respiratory Syntactical Virus (RSV).

All patients received a chest CT scan before ICU admittance with typical signs for COVID-19 pneumonia but no specific signs for IPA. Results of portable chest x-ray controlls during ICU-stay revealed only unspecific infiltrates. Broad-spectrum antibiotics were initiated in all critically ill patients. Standardized laboratory parameters according to the study protocol included C reactive protein, procalcitonin, interleukin 6, lactat dehydrogenases, d-Dimer, leukocyte coount, and lymphocyte count. No statistical significance could be observed between COVID-19 patients with and without CAPA except for Interleukin-6 (median 259, range 28–793 versus median 118, range 12–234; p = 0.013). Laboratory parameters for CAPA survivors and non-survivors are listed in Table 2.

**Table 2:** Characteristics of CAPA survivors and non-survivors.

	CAPA Survivors (N = 7)	CAPA Non-Survivors (N = 4)	p value
**Baseline and ICU characteristics**			
Mean age, years (SD)	74±8	70±8	0.889
Male sex (n, %)	5 (71)	2 (50)	0.413
Mean APACHE II (SD)	21±2	24±2	0.134
Mean SOFA (SD)	11±1	14±1	**0.026**
Median days of ICU stay (IQR)	23 (14–27)	17 (12–22)	0.276
Mechanical Ventilation days (IQR)	21 (11–25)	17 (12–22)	0.089
**Diagnostics**			
Positive NBL galactomannan	7	4	-
Positive serum galactomannan	0	4	-
NBL culture positive (n)	5	4	-
- *Asp*. *fumigatus*	5	4	-
AspICU criteria			
- proven	0	0	
- putative	7	4	
Mean Days from Intubation till CAPA diagnostic (IQR)	4 (1–7)	4 (1–7)	
Mean antimycotic treatment days (SD)	21±3	17±4	
Initial treatment (n)			
- Voriconazole	4	1	
- Isavuconazole	0	1	
- Liposomal amphotericin B	3	2	
Median NBL GM (IQR):			
- Day 1	4.6 (4.6–6.7)	6.3 (6.3–7.4)	0.182
- Day 3	2.5 (1.8–6.4)	3.5 (1.8–6.1)	0.958
- Day 7	5.1 (3.8–7.4)	4.5 (2.8–6.2)	0.682
- Day 10	3.1 (1.6–4.4)	4.3 (1.2–5.8)	0.928
- Day 14	2.8 (1.2–4.8)	5.7 (5.6–5.8)	0.118
- Day 17	2.4 (2.1–3.5)	2.9 (2.9–3.2)	0.976
- Day 20	2.5 (1.5–3.5)	5.5 (5.5–6.7)	0.097
- Day 23	2.5 (1.5–3.5)	-	-
- Day 27	<0.5	-	-
Mean Serum GM			
- Day 1	<0.5	1.5	-
- Day 3	<0.5	1.2	-
- Day 7	<0.5	1.5	-
- Day 10	<0.5	0.8	-
- Day 14	<0.5	1.3	-
- Day 17	<0.5	0.8	-
- Day 20	<0.5	-	-
- Day 23	<0.5	-	-
- Day 27	<0.5	-	
SARS-CoV-2 PCR positive at diagnosis of CAPA (n)	7	4	
Mean SARS-CoV-2 PCR positive days (IQR)	12 (9–20)	17	**<0.001**
Number of patients with SARS-CoV-2 antibodies	7	0	**<0.001**
Initial Horowitz index and follow up after initiation of antimycotics:			
-Day 1	118	77	**0.036**
-Day 3	145	91	**0.030**
-Day 7	190	104	**0.048**
Interleukin 6 (SD):			
- Day 1	344±248	228±195	0.659
- Day 3	346±323	1453±688	**0.006**
- Day 7	174±136	1970±824	**0.002**
- Day 10	142±65	1119±815	**<0.001**
- Day 14	164±56	1001±567	**0.038**
- Day 17	83±57	189±24	0.080
- Day 20	70±8	345±0	**0.034**
- Day 23	80±18	-	-
- Day 27	65±43	-	-
LDH (SD):			
- Day 1	529±261	637±462	0.243
- Day 3	388±120	480±90	0.777
- Day 7	331±99	1104±278	**0.004**
- Day 10	294±54	376±160	**0.012**
- Day 14	341±76	415±129	0.260
- Day 17	311±60	281±0	0.711
- Day 20	274±16	1540±-0	0.645
- Day 23	242±50	-	-
- Day 27	213±30	-	-

COVID-19 asscociated invasive pulmonary aspergillosis (CAPA); bronchial aspirates (BA); acute physiology and chronic health evaluation (APACHE II); sequential organ failure assessment (SOFA); galactomannan (GM); lactat dehydrogenases (LDH).

In total, 11 (34%) of 32 critically ill COVID-19 patients fulfilled the modified invasive pulmonary aspergillosis definition for putative invasive pulmonray aspergillosis (see above). Three patients did not meet the modified criteria; these were defined as colonised and excluded from the study. Median time till diagnosis of invasive pulmonary aspergillosis was 4 days (range 1–7) after ICU admission and intubation.

In the COVID-19 Cohort, mean age (mean: 72 versus 60 years; p = 0.003), APACHE II score (mean: 23 versus 17; p = 0.027) and ICU mortality were higher in patients with CAPA than in patients without CAPA (36% versus 9.5%; p<0.001).

ICU stay (21 days (range 9–28) versus 17 days (range 5–28); p = 0.340) and mechanical ventilation days (20 days (range 8–28) versus 15 days (range 3–28); p = 0.570) were not different between COVID-19 patients with and without CAPA.

Dividing the COVID-19 cohort into patients of CAPA survivors (n = 7) and CAPA non-survivors (n = 4) the following differences could be observed:

SOFA score (mean: 11 versus 14; p = 0.026), intially Horowitz Index and follow up Horrowitz Index after antimycotic initiation were significantly different for CAPA non survivors as compared to CAPA survivors (day 1: 77 versus 118; p = 0.036; day 3: 91 versus 145; p = 0.030; day 7: 104 versus 190; p = 0.048). Mechanical ventilation days (21 days (range 11–25) versus 17 days (range 12–22); p = 0.089) were not significantly different.

In 9 (82%) of the 11 patients with IPA, cultural growth of *Aspergillus spp*. could be established revealing *Asp*. *fumigatus* in all cases. GM- ODI in all NBL specimes of CAPA patients was >1 in repeated measurements. Positive serum GM could only be observed in the CAPA non-survivors group. All patients received antimycotic therapy. Therapy monitoring using NBL GM revealed decreasing GM levels during observation time in the CAPA survivors but not in the CAPA non survivors (see Table 2).

The definition of IPA used in this study is based on nondirected bronchial lavage Glactomannan testing with a cutoff of 1 optical density index, for which a sensitivity and specificity of 95% and 87%, respectively, could be calculated. For the cultural proceedings a sensitivity and specificity of 82% and 100%, respectively, and for the the serum Galctomannan a sensitivity and specificity of 36% and 100% could be found in our study collective.

SARS-CoV-2 PCR was positive in all CAPA patients at ICU admittance. Mean days of SARS CoV-2 positivity were significantly shorter in CAPA survivors than in CAPA non survivors (12 (range 9–20) versus 17 days; p<0.001). None of the CAPA non survivor patients developed antibodies against SARS CoV-2 in contrast to all CAPA surviors (p<0.001).

After 28 days significantly more patients without CAPA were discharged from the hospital (8 versus 1; p = 0.025), no differences could be observed between ICU and general ward stay at day 28 (see Table 1).

In the COVID-19 cohort 11 (34%) were diagnosed with IPA in contrast to 5 (8%) out of 64 patients in the COVID negative control cohort (see Table 3).

**Table 3:** Baseline characteristics of the COVID-19 negative control cohort.

	COVID negative Cohort (n = 64)	Without IPA (n = 59)	With IPA (n = 5)	p value
**Baseline characteristics**				
Mean age, years (SD)	68±15	68±16	58±12	0.154
Male sex (%)	44 (65)	40 (65)	4 (80)	0.160
Mean APACHE II (SD)	20±3	19±2	25±4	**<0.001**
Mean SOFA (SD)	10±2	10±2	13±2	**0.002**
**Risk factors**				
**n (%)**				
COPD	13 (20)	11 (18)	2(40)	0.119
Asthma	5 (7,5)	5(8)	3(60)	0.145
DM type 2	22 (33)	22 (36)	5 (100)	**<0.001**
Art. hypertension	25 (37)	24 (39)	1 (20)	**0.003**
Coronary heart disease	32 (50)	31 (50)	1 (20)	**<0.001**
CKD	19(38)	16 (26)	3 (60)	0.368
Atrial fibibrillation	3 (5)	3(5)	4 (80)	0.286
Immunosuppression	0	0	0	
Liver cirrhosis	13(20)	11 (18)	2 (40)	0.119
Pancreatitis	9 (14)	8 (13)	1 (20)	0.470
**ICU data**				
Mechanical ventilation (n)	64	59	5	0.156
Mechanical Ventilation days (IQR)	17 (7–38)	16 (6–38)	21(7–29)	0.266
Need for vasopressors (n,%)	64 (100%)	59 (100%)	5	0.123
Renal replacement (n,%)	25/64 (39)	20/59 (34)	5/5 (100)	**<0.001**
**Outcome data**				
Median days of ICU stay (IQR)	18 (7–38)	18 (7–38)	23 (7–35)	0.190
ICU mortality (n, %)	37 (55)	34 (55)	3 (60)	0.820
**Diagnostics**				
Median BAL Galactomannan (range)		<0.5	2.9 (1.8–6.4)	
Median serum galctomannan (range)		<0.5	0.9 (0.7–1.5)	
BAL culture positive (n)			3	
AspICU criteria (n)				
- Proven			0	
- Putative			5	
- Coloniser			0	
*Asp*. *fumigatus*			3	

Abbreviations: Invasive pulmonary aspergillosis (IPA); acute physiology and chronic health evaluation (APACHE II); sequential organ failure assessment (SOFA); chronic obstructive pulmonary disease (COPD); diabetes mellitus type 2; chronic kidney disease; intensive care unit (ICU), broncho alveolar lavage (BAL).

The basic characteristics of both groups are presented in Table 4.

**Table 4:** Patient characteristics of COVID-19 and the control cohort.

	COVID-19 cohort (n = 32)	Control cohort (n = 64)	p value
**Baseline characteristics**			
Mean age, years (SD)	65±15	68±15	0.372
Male sex (%)	23 (72)	44 (65)	0.753
Mean APACHE II (SD)	18±4	20±3	0.103
Mean SOFA (SD)	10±3	10±2	0.466
**Comorbidities**			
**n (%)**			
COPD	3 (10)	13 (20)	0.175
Asthma	1 (3)	5 (7,5)	0.660
DM type 2	8 (25)	22 (33)	0.350
Art. hypertension	21 (65)	25 (37)	**0.014**
Coronary heart disease	2 (6)	32 (50)	**<0.001**
CKD	5 (16)	19(38)	0.134
**ICU data**			
Mechanical Ventilation days (range)	16 (3–28)	17 (7–38)	0.889
Median days of ICU stay (IQR)	18 (5–28)	18 (7–38)	0.929
ICU mortality (n, %)	6 (19)	37 (55)	**<0.001**

Acute physiology and chronic health evaluation (APACHE II); sequential organ failure assessment (SOFA); chronic obstructive pulmonary disease (COPD); diabetes mellitus type 2; chronic kidney disease; intensive care unit (ICU).

In the total patient cohort including the negative controls COVID-19 (11/16 versus 21/80, p = 0.001), higher APACHE II score (18.7±3.1 versus 23.0±3.1; p<0.001), higher SOFA score (median 10, range 4–17 versus median 12, range 10–15; p<0.001) and coronary heart disease (2/16 versus 32/80, p = 0.036) were associated with IPA.

**Fig 2:**
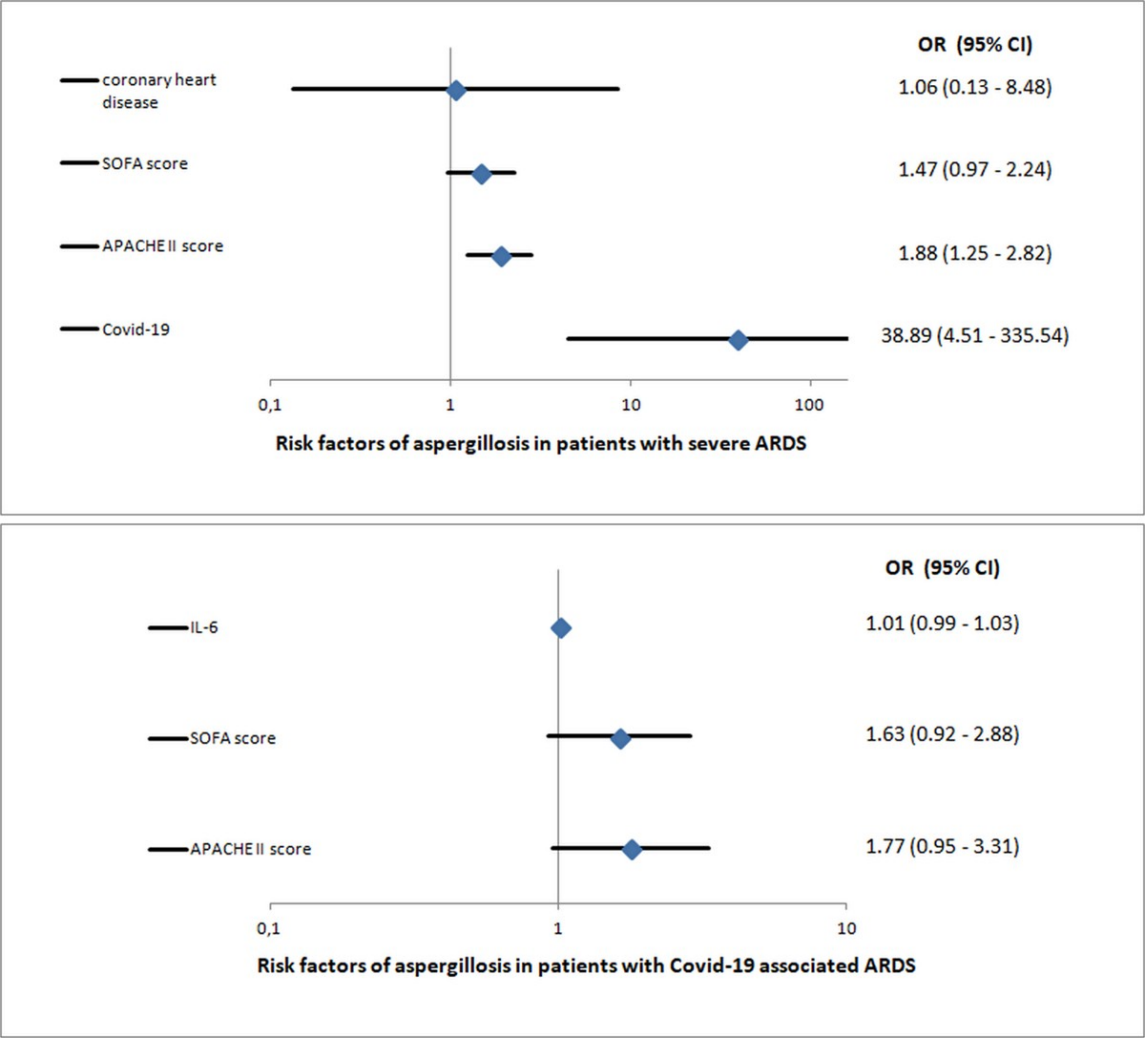
Results of regression models displayed by Forest Blots: A) Risk factors of invasive pulmonary aspergillosis in total patient cohort (COVID-19 patients and controls); B) Risk factors of invasive pulmonary aspergillosis in patients with COVID-19 (OR: odds ratio; 95% CI: 95% confidence interval; IL-6: interleukin -6).

To assess whether COVID-19 was independently associated with IPA, a binary logistic regression analysis was performed. This analysis confirmed an independent association between COVID-19 and IPA. Also a higher APACHE II score was independently associated with IPA (Fig 2A).

Regarding the COVID-19 cohort, none of the univariate significant parameters were independently associated with IPA in the logistic regression analysis (Fig 2B).

Details of the regression models are presented in S1 Table.

## Discussion

In our prospective study we aimed to gain data on incidence, risk factors and outcome of IPA in critically ill patients with severe COVID-19 pneumonia using a standardized screening protocol. Moreover, our study shows, that COVID-19 is an independent risk factor for IPA.

In comparison to our control cohort, COVID-19 increased the risk of developing an IPA from 8% to 34% and CAPA is associated with a mortality rate of 36%. These findings are as high as previously shown mortality rates, espcially reported from patients with severe influenza pneumonia and is in the line with other reported CAPA studies [9–11].

Although, typical risk factors for COVID-19 e.g. arterial hypertension, diabetes mellitus type 2 or coronary heart disease were also significantly more frequent in our COVID-19 cohort, most likely based on the limited number of patients in the CAPA cohort, these risk factors were not associated with an increased risk of developing CAPA [12].

Moreover, none of these patients fulfill the typical EORTC/MSG host factor criteria, and only a few COVID-19 patients (4/32) had a history of a chronic pulmonary disease prior to the infection with SARS-CoV-2.

In fact, most patients with COVID-19 presented with mild flu-like symptoms, but up to 15% of the affected patients required assisted oxygenation and 5% of them deteriorated towards a severe ARDS as presented in our study cohort [12]. Data on intrinsic risk factors which may predispose to severe ARDS- in COVID-19 patients are sparse. Only small pathological studies of patients with severe COVID-19 associated ARDS, report a typical diffuse alveolar damage combined with intra-alveolar neutrophilic infiltration and vascular congestion, which is interpreted as an acute phase component [13, 14]. Therefore, SARS-CoV-2 might trigger an imbalanced immune response resulting in a ‘cytokine stom’ and extensive pulmonary inflammation [15]. If the proposed mechanism causing lung injury is a consequence of the described pathological findings or vice versa has to be investigated in further studies. Taken together, it could be assumed that these findings, similary reported in SARS, may lead to an impaired mucociliary activity stimulated by immune cell dysfunction and immune system dysregulation which paves the way for secondary infections [13, 15].

It has been shown in several studies including ours, that these super-infections have a negative impact on the outcome of affected patients. [16, 17]. The overall incidence of IPA is in line with reported rates for critically ill patients varying between 1 and 7% [16, 17]. Furthernore our data increases the awareness of IAP as complication in patients with COVID-19 associated ARDS and helps clinicians to establish standardized screening methods for invasive pulmonary aspergillosis and to early identify high risk patients.

In this study we used the modified AspICU score and CAPA definitions for diagnosis of CAPA in combination with standardized time intervals of screening (see above) [9–11]. As clinical criteria such as ongoing fever, dyspnea or worsening respiratory insufficiency are also typical of COVID-19 and radiological findings in non-neutropenic patients in most cases do not allow to discrimante typical mycological findings from COVID-19, the diagnosis of CAPA is mostly based on mycological criterias [10, 18, 19].

GM-detection in BAL is a valid test to confirm or rule out IPA with a sensitivity and specificity of approximatley 90% using an ODI-cut-off of ≥0.8 [20]. Based on our datas and the underlying IPA definitions used in this setting a sensitivity and specificity of 95% and 87%, respectively, for NBL GM testing could be calculated.

Due to safety concerns regarding—Link kopiertaerosolization and surface stability of SARS-CoV-2, only nondirected bronchial lavage (NBL) from deep bronchial suction via a closed system were used for diagnostics as recommend by ESCIM guidelines [21, 22]. Thus, NBL is not validated for GM detection. Although, NBL is also used in other CAPA studies, however, if the mentioned ODI cut-off for BAL is also reliable for NBL specimenis needs further investigation [9–11]. Moreover, increasing the ODI cut-off does not necessarily increase the sensitivity and specificity as reported in some studies [23].

Therefore, as recommended and used in other studies we used a galactomannan ODI cut-off of >1 for NBL equivalent to BAL specimens. Following these considerations we found putative IPA in 34% of our critically ill COVID-19 patients, which is nearly a similar rate to what has been observed in patients with severe influenza pneumonia and also in recent CAPA studies [9–11, 24].

Another problem which is also reported in other studies using NBL lays in the nature of undirected sampling itself. Although, patients with severe COVID-19 pneumonia presenting consolidation in all lung areas, it is not known which of these are caused by SARS CoV-2 or by fungals [18]. These circumstances could lead to a potentially overdiagnosis of IPA. In contrast, in our study, the median NBL GM ODI of all patients within the CAPA cohort was 5.4 (range 1.8–7.4). Moreover, cultural growth of *Aspergillus spp*. in 82% of NBL specimens of the CAPA patients together with serum GM-detection with ODI >0.5 in 4 CAPA patients strongly emphazise that CAPA is a relevant complication in severe COVID-19 pneumonia.

Also in the light of the new consensus criteria for CAPA diagnosis, recently published by Koehler et al., our results are in the line with the new definitions [25].

Moreover, as also stated in these guidelines further studies are needed to evaluate the potential of NBL not only in COVID-19 patients [25].

Mean timepoint of CAPA diagnosis was day 4 of ICU stay, which seems early. This may be explained by the fact that all patients already have had reported severe COVID-19 symptoms for several days prior to hospital admission. This observation is in the line with other studies [9–11, 26]. Antimycotic therapy was initiated in all patients with putative IPA according to recent guidelines [27]. The decreasing NBL galactomannan levels and increasing Horovitz indexes during therapy suggest an effect of the specific antmycotics beside general ARDS management in the CAPA survivors group. Limited to the small sample size the concrete effect of antifungal therapy regarding outome improvement can not be drawn from our study. Moreover, referring to the high observed incidence of CAPA, the role of antifungal prophylaxis should be further studied.

Based on our findings, there was no difference in median ventilation and ICU days between the COVID-19 cohort with and without CAPA, which may be explained by the severity of COVID-19 itself. CAPA patients were significantly older, had a higher APACHE II score and a higher mortality rate as mentioned above.

However, some findings in the CAPA non survivors group were striking. Increased serum interleukin-6 and LDH levels could be associated with worse outcome in COVID-19 patients [28]. These findings could be confirmed in the CAPA non-survivors with significantly elevated serum levels, especially of interleukin 6. Moreover, the role of SARS-CoV-2 not only in the pathophysiology of lung injury but also in enhancing an ongoing infection have to be investigated in further studies. In our cohort all CAPA survivors cleared the detectable SARS-CoV-2 RNA and generated IgG antibodies during their ICU stay in contrast to CAPA non survivors with virus persistence until death.

Our study had some limitations. First, it is a single center experience with a limited number of COVID-19 patients and, confounding cannot be ruled out. However, we prospectively studied a standardized screening tool in patients with severe COVID-19 pneumonie in comparison to a retrospective control cohort.

Secondly, the usage of NBL instead of BAL is a new approach and confirms the experiences from other studies [9–11]. Given the observations in our study cohort, showing that diagnosis was confirmed in several follow up examinations and by cultures, as well as the special circumstances in COVID-19 patients, we believe, that NBL in mechanical ventilated patients gained through deep bronchial suction, are a suitable alternative for GM-testing. However, comparing NBL with BAL from control cohort patients may be a confounder.

Finaly, due to the novelty of COVID-19 there is still limited information about pathophysiology and clinical characteristics particularly in critically ill patients. Therfore, further studies are needed to analyse out risk profiles for development of CAPA.

In conclucion, in critically ill COVID-19 patients, Covid-19 associated invasive pulmonary aspergillosis is highly prevalent and associated with a high mortality rate. COVID-19 and a high APACHE II score are independently associated with invasive pulmonary aspergillosis. A standrrdized screening and diagnostic approach as presented in our study can help to identify out affected patients at an early stage.

## Supporting information

S1 Table Details of regression models with IPA as dependent variable in the total patient cohort and the COVID-19 positive patient cohort. (DOCX)
