# Supplementary material for: Invasive pulmonary aspergillosis in critically ill patients with severe COVID-19 pneumonia: Results from the prospective AspCOVID-19 study
Source: PLoS One. 2021 Mar 17;16(3):e0238825. doi: 10.1371/journal.pone.0238825 (PMC7968651; doi:10.1371/journal.pone.0238825)
Supplement: S1 Table — (DOCX) [file pone.0238825.s001.docx]

S1 Table. Details of regression models with IPA as dependent variable in the total patient cohort and the COVID-19 positive patient cohort.

| Total patient cohort including COVID-19 positive and negative patients (n=96) | | | | |
| --- | --- | --- | --- | --- |
| Independent variable | p in unvariate analysis | Exp(B) | 95% CI | p in regression analysis |
| COVID-19 | 0.001 | 38.9 | 4.5 – 335.5 | 0.001 |
| APACHE II score | <0.001 | 1.9 | 1.3 – 2.8 | 0.002 |
| SOFA score | <0.001 | 1.5 | 1.0 – 2.8 | 0.07 |
| Coronary heart disease | 0.036 | 1.1 | 0.1 – 8.5 | 1.0 |
|  |  |  |  |  |
| COVID-19 positive patient cohort (n=32) | | | | |
| Independent variable | p in unvariate analysis | Exp(B) | 95% CI | p in regression analysis |
| APACHE II score | <0.001 | 1.8 | 1.0 – 3.3 | 0.072 |
| SOFA score | 0.003 | 1.6 | 0.9 – 2.9 | 1.0 |
| IL-6 at ICU admission | 0.012 | 1.0 | 0.99 – 1.03 | 0.248 |
|  |  |  |  |  |
